# Feed Restriction Modulates Growth, Gut Morphology and Gene Expression in Zebrafish

**DOI:** 10.3390/ijms22041814

**Published:** 2021-02-11

**Authors:** Kathiresan Purushothaman, Jerryl Kim Han Tan, Doreen Lau, Jolly M. Saju, Natascha M. Thevasagayam, Caroline Lei Wee, Shubha Vij

**Affiliations:** 1 Reproductive Genomics Group, Temasek Life Sciences Laboratory, Singapore 117604, Singapore; kathiresan.purushothaman@nord.no (K.P.); doreen.lau.a.h@gmail.com (D.L.); jolly@tll.org.sg (J.M.S.); natmay@gmail.com (N.M.T.); 2 Institute of Molecular and Cell Biology, 61 Biopolis Dr, Singapore 138673, Singapore; 18026184@myrp.edu.sg

**Keywords:** *Danio rerio*, gut, calorie restriction, feed restriction

## Abstract

A reduction in daily caloric or nutrient intake has been observed to promote health benefits in mammals and other vertebrates. Feed Restriction (FR), whereby the overall food intake of the organism is reduced, has been explored as a method to improve metabolic and immune health, as well as to optimize productivity in farming. However, less is known regarding the molecular and physiological consequences of FR. Using the model organism, *Danio rerio*, we investigated the impact of a short-term (month-long) FR on growth, gut morphology and gene expression. Our data suggest that FR has minimal effects on the average growth rates, but it may affect weight and size heterogeneity in a sex-dependent manner. In the gut, we observed a significant reduction in gut circumference and generally lower mucosal heights, whereas other parameters remained unchanged. Gene Ontology (GO), EuKaryotic Orthologous Groups (KOG), and Kyoto Encyclopedia of Genes and Genomes (KEGG) analysis identified numerous metabolic, reproductive, and immune response pathways that were affected by FR. These results broaden our understanding of FR and contribute towards growing knowledge of its effects on vertebrate health.

## 1. Introduction

Feed Restriction (FR) refers to a feeding regimen, wherein the nutrient and calorie intake of an organism is reduced by decreasing the ration size, feeding time, or feeding duration [1]. This could include the complete cessation of feeding for a period of time, followed by subsequent refeeding [2,3,4,5,6,7,8].

A similar feeding regimen that is known as Calorie Restriction (CR) involves restricting daily energy intake by ~20–50%, albeit without the deprivation of essential nutrients or malnutrition [9,10,11,12,13]. Given that many studies in the literature define CR as a general reduction in total food intake, rather than of calories alone (e.g., Mattison et al., 2003, Ma et al., 2020), we will refer to these CR studies more broadly as FR throughout the rest of this manuscript.

FR has been touted to have a positive impact on health, ageing, and lifespan, although mixed effects have been observed in both animal and human studies. For example, it has been associated with oxidative stress inhibition, decreased cancer risk, and reduced inflammation in mammalian models [11,14], as well as higher intestinal nutrient uptake and absorptive capacities [15]. Additionally, feed-restricted male mice have been shown to have increased insulin sensitivity, reduced low-density lipoprotein and cholesterol levels, as well as improved physical performance [13]. A few studies have demonstrated the favorable impact of FR on both innate and acquired immunity in mice, rats, and adult humans [16,17]. Furthermore, FR (30–70% of their normal calorie consumption) has been shown to increase the lifespan in both invertebrate and vertebrate models, with potential implications in protecting humans from cardiovascular disease, cancer, and many other health problems [13]. A reduction in feeding has also been shown to positively affect the metabolic rates, growth, survival, and reproductive fitness of fish [18].

Notably, in the majority of mammalian studies, FR results in a loss of fat and body mass [19,20,21,22]. Furthermore, some studies have observed the detrimental effects of FR, such as blunted muscle protein synthesis in humans [23]. Hence, the costs and benefits of feed restriction on vertebrate health still need to be better understood.

FR is also a strategy often employed in agriculture and aquaculture. The advantages of restricted feeding in farmed animals include improved nutrient utilization, compensatory growth, and a lowered production cost [8,24]. For example, in poultry, FR has been observed to reduce the risk of metabolic disease and death, in addition to improving feed conversion [1]. In the catfish, *Ictalurus punctatus,* long-term FR during winter months enhanced resistance to disease in older animals [4,5,6,7]. At the same time, catfish that were not fed during the winter months showed no significant difference in their survival, weight gain, and feed conversion after they were fed again in spring, indicating efficient compensatory growth [4,5].

A study on restricted feeding in Asian seabass (*Lates calcarifer*) also established that there was no difference in the mean weight, growth rate, or protein efficiency ratio for fish undergoing starvation and refeeding cycles, as compared to fish fed to satiation [25]. Notably, another study on Asian Seabass showed that a 1–3 week starvation induced significant changes in gut histology, such as lower mucosal height, goblet cell numbers, and muscularis thickness [26]. In European seabass (*Dicentrarchus labrax)*, 50% feed restriction did not significantly affect fish weight or filet yield, although the muscle lipid content was lower in the restricted fish [27]. Hence, there are likely both species and protocol-specific differences in the effects of FR on animal growth, health, and metabolic physiology.

In our study, we used zebrafish (*Danio rerio)* to model the effects of FR on teleost fish. With a fully sequenced genome, short generation interval, and conserved organs and systems, it is a useful model organism for nutrigenomics and nutrigenetics research [28]. Some previous studies have examined the effects of FR on zebrafish gene expression and physiology. One such study showed a decrease in the expression of 466 transcripts and increased expression of 108 transcripts in the livers of 21 days-starved zebrafish [29]. This study also reported a significant decrease in liver expression of genes involved in fatty acid biosynthesis and protein metabolism. However, starvation had little to no effect on the brain transcriptome [29]. In another study, feed-restricted juvenile zebrafish were found to prioritize scale formation over somatic growth, whereas sexually matured adults prioritized reproduction over somatic growth, and somatic growth over fat storage [30]. 

The gut plays a crucial role in the maintenance of fish health, nutrient regulation and growth, as well as the host immunity to pathogens [31,32,33,34]. Feed intake can have a direct impact on normal gut homeostasis. Currently, there is limited information and research regarding the effects of feed restriction on zebrafish intestinal physiology. A comprehensive analysis of specific genes and metabolic pathways that can be affected by feed restriction in the gut are currently scarce.

In this study, we examined the effects of 85% and 70% FR (i.e., an 85% or 70% ration) on the growth, intestinal morphology, and intestinal gene expression of three-month post fertilisation (mpf) zebrafish (AB strain). FR did not drastically affect the average growth rate of zebrafish, although size heterogeneity was increased in male zebrafish under 70% feeding, and occasional size differences were observed in females. FR also significantly reduced the intestinal circumference and appeared to reduce mucosal height, although other aspects of gut morphology remained unchanged. Finally, FR led to the upregulation of various metabolic and immune pathways, as well as the suppression of reproduction-related gene expression. Our results reveal both the potential benefits and downsides of moderate feed restriction, which may inform both farming practices and human health research.

## 2. Materials and Methods

### 2.1. Ethics

All of the experimental procedures involving the animals were performed according to the Responsible Use and Care of Laboratory Animal course guidelines, under ethics approval by the Institutional Animal Care and Use Committee (IACUC) of Temasek Life Sciences Laboratory. The experiments were conducted at Temasek Life Sciences Laboratory (TLL) and they were approved by the TLL Institutional Animal Care and Use Committee (approval ID: TLL (F)-14-002, Approval date: 23 January 2014).

### 2.2. Fish Husbandry and Feeding Strategy

Inbred adult zebrafish of wild-type AB strain were raised and maintained according to the standard protocol. The fish were reared in AHAB recirculation systems (Aquatic Habitats, Pentair AES, NC, USA) at an ambient temperature (26–28 °C), pH (7.0–7.5), and photoperiod of 14/10 h light/dark cycle.

Embryos (400–500) that were produced from a mass cross were fed rotifers (Aquatic Biosystems, Fort Collins, CO, USA) beginning at five days-post-fertilisation (dpf), followed by artemia (Aquafauna Bio-Marine, Hawthorne, CA, USA) beginning at 14 dpf, and then advanced to feeding on TetraMin tropical fish food flakes (Tetra Werke, Melle, Germany) at 28 dpf. The TetraMin tropical fish food flakes were distributed by hand to all adult zebrafish twice a day, feeding to 100% visual satiety from both the water surface and water column. Excess or leftover feed were siphoned away immediately after each feeding session. Beginning two months-post-fertilisation (mpf), zebrafish of uniform size were stocked in groups of 45 in 9-L recirculating tanks. At this age, sex is still difficult to determine and, thus, the fish were not sorted by sex. Each week, weights were obtained from a random set of approximately 30 zebrafish from the same batch, and sex confirmed post-hoc by gonad dissection. The amount of feed to be given for 100% satiety was re-calculated weekly based on 3% of the total fish body weight (number of remaining fish × average weight) and feeding completion within 5 min. The fish were fed with freshwater fish feed, TOMBOY Micro 80 (Skretting, Nutreco, Norway) to 100% satiety consecutively for four weeks (acclimatization period) before beginning the FR experiment. Feeding was done twice a day (9 a.m. and 4 p.m.; Monday–Sunday).

The fish feed, TOMBOY Micro 80 (Skretting, Nutreco, Norway), was in the form of floating micro pellets composed of crude protein (42%), crude fats (6%), ash (11%), fiber (3%), and moisture (11%). According to the supplier’s manual, the ingredient list contained plant-based components, such as soybean oil, soy lecithin, corn gluten meal, wheat, rice, vegetal concentrate, fish-based, and other marine sources, such as steam dried fishmeal, fish oil, squid meal, seaweed meal, as well as spirulina, vitamins, minerals, amino acids, yeast cell walls, mannan oligosaccharides, beta-glucan, acidifiers, and essential oils.

### 2.3. Feed Restriction Experiment and Sample Collection

**Figure 1:**
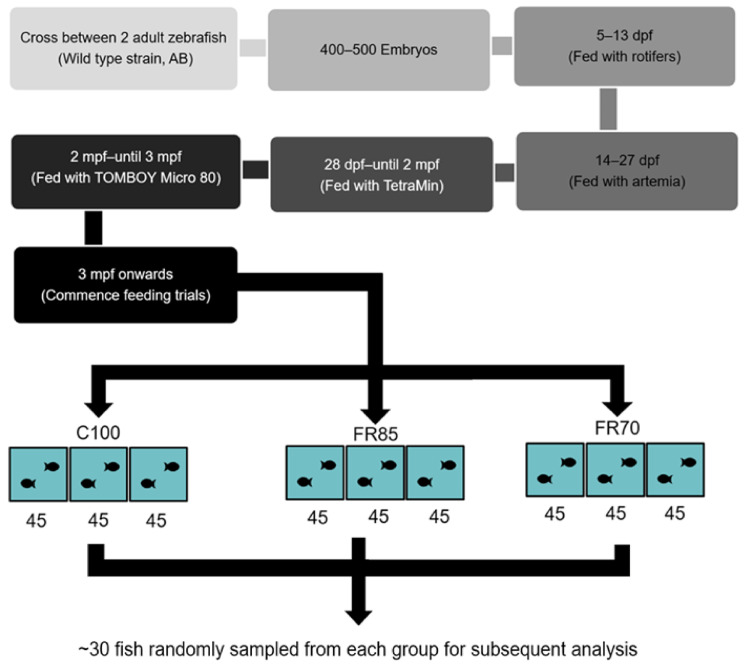
Flow chart outlining the experimental design for assessing the effect of feed restriction on zebrafish. Abbreviations: dpf: days-post-fertilization; mpf: months-post-fertilization; C100, FR85, and FR70 refer to feeding the fish to 100%, 85%, and 70% satiety, respectively; 45 refers to the number of zebrafish stocked in each experimental tank. Approximately 30 fish were sampled each week for analysis.

At 3 mpf, the fish were divided into three treatment groups: 100% feeding, 85% feeding, and 70% feeding (fed with commercial fish feed). These three groups of fish were classified as Group 1: 70% fed group (FR70), Group 2: 85% fed group (FR85), and Group 3: 100% fed groups (C100). Each treatment group consisted of three tanks of 45 zebrafish each. FR was performed over a period of four weeks (Figure 1).

At each sampling point, approximately 30 fish (which had not been fed since the prior feeding time) were sampled from each group and then euthanized by cold ice treatment. For growth determination, the weights (mg) of individual sampled fish from each group were recorded before sacrificing the fish to obtain tissue samples for analyses.

For RNA extraction, five pools (~five fish each) of the mid-intestine were generated from each of the three groups on week 4 (C100: 15 males, 10 females; FR85: 15 males, eight females; FR70: 15 males, seven females). A previous study [35] describing the division of the zebrafish intestine into the anterior, middle, and posterior segments was used as a reference to identify the mid-intestine in this study. Equal amounts of mid-intestine were collected from each fish within a pool. The samples were immediately placed in liquid nitrogen and stored at −80 °C until further use. 

### 2.4. Histology and Microscopy

After 42 h of fixation in 10% chilled formalin solution, the intestines were dehydrated in increasing gradients of ethanol (50%, 65%, 75%, 85%, 95%, and 100%). The samples were embedded in hydroxyethyl methacrylate (Historesin mounting medium, Leica, Germany) for three days. A series of ~5–10 histological sections were performed on each sample (section thickness ~5 µm), mounted on slides, and stained using Haematoxylin and Eosin (H&E; Sigma Aldrich, St. Louis, MO, USA). Image acquisition was performed using a Zeiss Axioplan 2 microscope mounted with a Nikon (Tokyo, Japan) digital camera DXM 1200F. The external circumference of the serosa, mucosal height, and muscularis layer thickness were quantified using ImageJ (Fiji) software, version IJ 1.46r, Wisconsin, USA, [36]. The number of goblet cells was determined by manually counting them in the complete mucosal region under microscope.

### 2.5. RNA Extraction, Library Construction and Sequencing

RNA extractions from the mid-intestine samples were performed according to the manufacturer’s instructions while using RNeasy Kit (Qiagen, Valencia, CA, USA). The RNA concentration and integrity of each sample were measured using a NanoDrop ND-1000 Spectrophotometer (NanoDrop Technologies, DE, USA) and an Agilent 2100 Bioanalyzer. The total RNA from the mid-intestine portion of 5 fish was used to prepare mRNA libraries for sequencing following the standard Illumina (CA, USA) protocol using TruSeq^®^ Stranded Total RNA Sample Preparation kit for low sample number. The multiplexed RNA-seq libraries (12X) were sequenced with a read length of 75 nucleotides using the Illumina Nextseq 500 system.

### 2.6. Mapping of Reads and Differential Gene Expression Analysis

Supplementary Table S1 provides the number of reads that were generated per sample along with the percentage of reads mapped to the zebrafish reference genome (danRer7) using Tophat v2.0.13. Cuffdiff v2.2.0 was used for the abundance calculation of transcripts that are represented as Fragments Per Kilobase of transcript per Million mapped reads (FPKM) and the identification of differentially expressed genes (DEGs). The criteria of at least two-fold difference, *p*-value < 0.05, and false discovery rate (FDR) < 0.1 was applied to identify the DEGs. The venn diagrams were created using the online tool, ‘Bioinformatics & Evolutionary Genomics’ (bioinformatics.psb.ugent.be/webtools/Venn (accessed on 25 May 2020).

### 2.7. Gene Ontology (GO), Eukaryotic Orthologous Groups (KOG) and Kyoto Encyclopedia of Genes and Genomes (KEGG) Analysis

For Gene Ontology (GO) analysis, UniProtKB IDs were used to obtain GO annotation, and functional classification was done using the online tool, ‘Panther classification system’ (Panther15.0, 21 February 2020; pantherdb.org/geneListAnalysis.do (accessed on 3 June 2020).

For functional analysis, we converted the gene names to UniProtKB IDs and subsequently converted to FASTA files by Uniprot, uniprot.org (accessed on 3 June 2020) and then submitted to EggNOG-mapper (EggNOG v5.0; eggnog-mapper.embl.de (accessed on 5 June 2020)) to obtain the Eukaryotic Orthologous Groups (KOG) annotations of differentially expressed genes. The KOG analysis was performed to classify the identified genes into four comprehensive classes: metabolism, cellular process and signaling, information storage and processing, and poorly characterized [37].

For Kyoto Encyclopedia of Genes and Genomes (KEGG) analysis, the FASTA files were uploaded to an online server, ‘KAAS–KEGG Automatic Annotation Server’ (genome.jp/kegg/kaas (accessed on 8 June 2020)) to obtain KEGG Orthology (KO) IDs. The KO IDs were then submitted to the KEGG mapper web server, http://www.genome.jp/kegg/tool/map_pathway2.html (accessed on 10 June 2020) for pathway analyses.

### 2.8. Statistics

**Figure 2:**
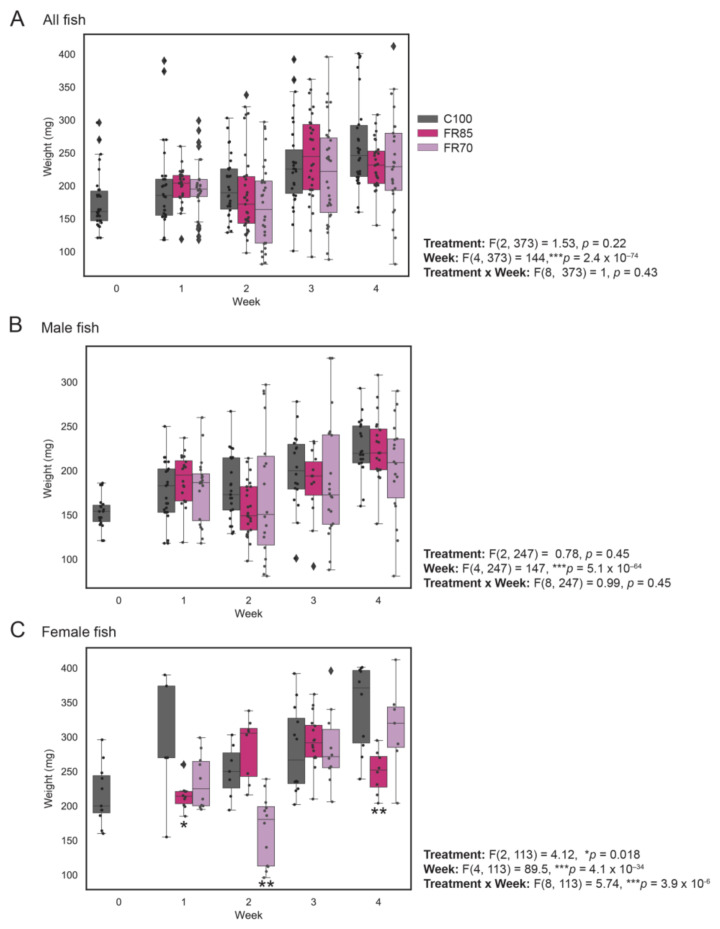
Weight profiles of zebrafish fed to visual satiety compared to feed-restricted fish (85% and 70% feeding). Feed restriction (85% and 70% feeding) was performed at 3 mpf and the weight measurements were recorded on a weekly basis for a period of 4 weeks. The combined weight of males and females (all fish), only males and only females are shown in (**A**–**C**), respectively. The box plot shows median, interquartile interval, and data range excluding outliers; each dot represents an individual fish. ♦ represents an outlier. Two-way ANOVA was used to infer the significant effects of treatment, week, or treatment by week interactions, followed by Tukey’s multiple comparison test to identify significantly different means. Means that are significantly different from Group 100% are noted with—* Padj < 0.05, ** Padj < 0.01.

**Figure 3:**
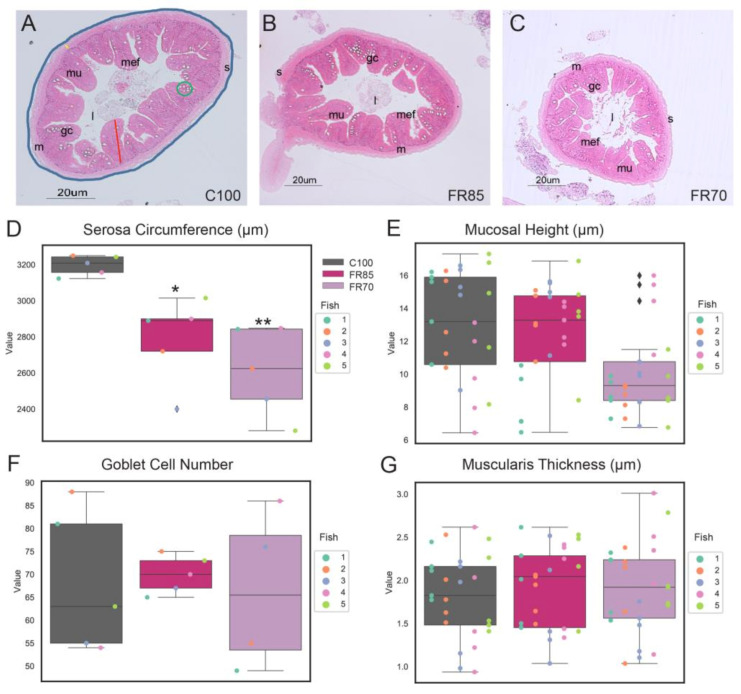
Histological analyses of feed restricted zebrafish reveal differences in some morphological characteristics. Representative transverse sections of Haematoxylin and Eosin (H&E)-stained mid-intestine from fish fed on (**A**) 100%, (**B**) 85%, and (**C**) 70% feed for a period of three weeks. The serosa circumference, mucosal height, muscularis thickness and goblet cell number are indicated by the blue line, red line, yellow line, and green circle, respectively. Scale bar = 20 µm. Abbreviations: serosa (s), lumen (l), mucosa (mu), muscularis (m), goblet cells (gc), and mucosal epithelial fold (mef). (**D**) External circumferences of serosa, (**E**) mucosal height, (**F**) goblet cell number, and (**G**) muscularis layer thickness were measured from sections of the midgut. The box plot shows median, interquartile interval, and data range excluding outliers; colored dots represent data from each individual fish. ♦ represents an outlier. In (**E**,**G**), multiple data points were sampled per fish. One-way ANOVA was performed between the mean of each group with the mean of the control group, followed by the Tukey Post-hoc test, adjusting for multiple comparisons. Means that are significantly different from C100 are noted with—* Padj < 0.05, ** Padj < 0.01.

One-way or two-way ANOVA, followed by the Tukey’s HSD Post-hoc test for multiple comparisons, was used to establish significance. * *p* < 0.05, ** *p* < 0.01, and *** *p* < 0.001. Statistical analysis and data visualization for Figure 2, Figure 3, and Figure S1 were performed using Python software (version 3.7).

## 3. Results

### 3.1. Weight Profiles of Fully Fed and Feed-Restricted Fish Revealed Minor Differences 

The average weights of approximately 30 fish were recorded weekly. Because sex is difficult to determine visually at this age, the sex ratios were only determined post-hoc via gonad dissection. There was an unequal number of male and female zebrafish at each sampling interval (Table 1). We also compared the body weights of males and females separately because females are known to be larger in size compared to male zebrafish at the same age. The combined weights of males and females (overall), only males, and only females across the three groups (100%, 85% and 70% feeding) are shown in Figure 2A–C, respectively (Figure 2 and Table 1).

All three groups of fish showed a significant increase in weight by the end of the four-week experiment (Figure 2A). Two-way ANOVA revealed no significant differences in the weights of the control and feed-restricted zebrafish over four weeks (F_treatment_(2, 373) = 1.53, *p* = 0.21), despite the largest average weight gain in control fish (50.39% increase, versus 32.87% for FR70 and 31.92% for FR85 fish).

Similarly, in male fish (Figure 2B), there were no significant differences in the weights of control and feed-restricted fish (F_treatment_(2, 247) = 0.78, *p* = 0.45, two-way ANOVA). However, particularly for male FR70 fish, size heterogeneity (i.e., variance in weights) appeared to be larger from Weeks 2–4, which was possibly due to competition for limited resources.

In contrast, we observed both a significant difference in the effect of treatment and a significant interaction between treatment and week (F_treatment_(2, 113) = 4.13, *p* = 0.018; F_treatmentxweek_(8, 113) = 5.74, *p* = 3.9 × 10^−6^; 2-way ANOVA) in female fish (Figure 2C), with a significant reduction in the FR85 female fish weights in Week 1 (*p* = 0.024) and Week 4 (*p* = 0.0045) and a significant reduction in FR70 female fish weights in Week 2 (*p* = 0.0023, Tukey Test). However, it is possible that random sampling and smaller sample sizes might have contributed to this variation.

### 3.2. Histological Analyses Reveal Differences in Some Morphological Characteristics

We next characterized the key components of the zebrafish’s mid-gut, such as its intestinal mucosal height, muscularis thickness, goblet cell numbers, and the external circumference of serosa (Figure 3).

Notably, the serosa circumference of feed-restricted guts (85% & 70%) was significantly lower than the controls (Figure 3A–D, F_treatment_(2, 12) = 11.2, FR70: *p* = 0.002, FR85: *p* = 0.02, One-way ANOVA and Tukey post-hoc test). The mean serosa circumferences were 2610 μm, 2785 μm, and 3196 μm for FR70, FR85, and C100 fish, respectively. The serosa circumference was not significantly different between FR70 and FR85 zebrafish (*p* = 0.38).

We observed no significant difference between the muscularis thickness (F_treatment_(2, 12) = 0.26, FR70: *p* = 0.85, FR85: *p* = 0.77; Figure 3G), the number of goblet cells (F_treatment_(2, 11) = 0.08, FR70: *p* = 0.9, FR85: *p* = 0.9; Figure 3F), and mucosal height (F_treatment_(2, 12) = 2.71, FR70: *p* = 0.11, FR85: *p* = 0.9; Figure 3E). However, the mucosal height in FR70 fish trended towards much lower values when compared to the other treatments (Figure 3E).

Given the observed sex differences in weight profiles, we further analyzed the gut morphology based on sex (Figure S1). Possibly due to the low sample size, we did not detect any clear sex-dependent differences in the effects of feed restriction, except that the reduction in gut circumference was more prominent in male fish (Figure S1A). Notably, we observed lower mucosal heights in male as compared to female zebrafish across all of the groups (Figure S1B).

### 3.3. RNA Sequencing-Based Transcriptomic Profiling

Total RNA was extracted from the mid-intestine samples, and RNA sequencing was performed, as described in Methods, in order to understand the effect of feed restriction on gut gene expression. RNA sequencing was performed on a mixture of males and females (similar ratios across all groups) sacrificed on Week 4 (see Section 2 and Supplementary Table S1 for details).

**Figure 4:**
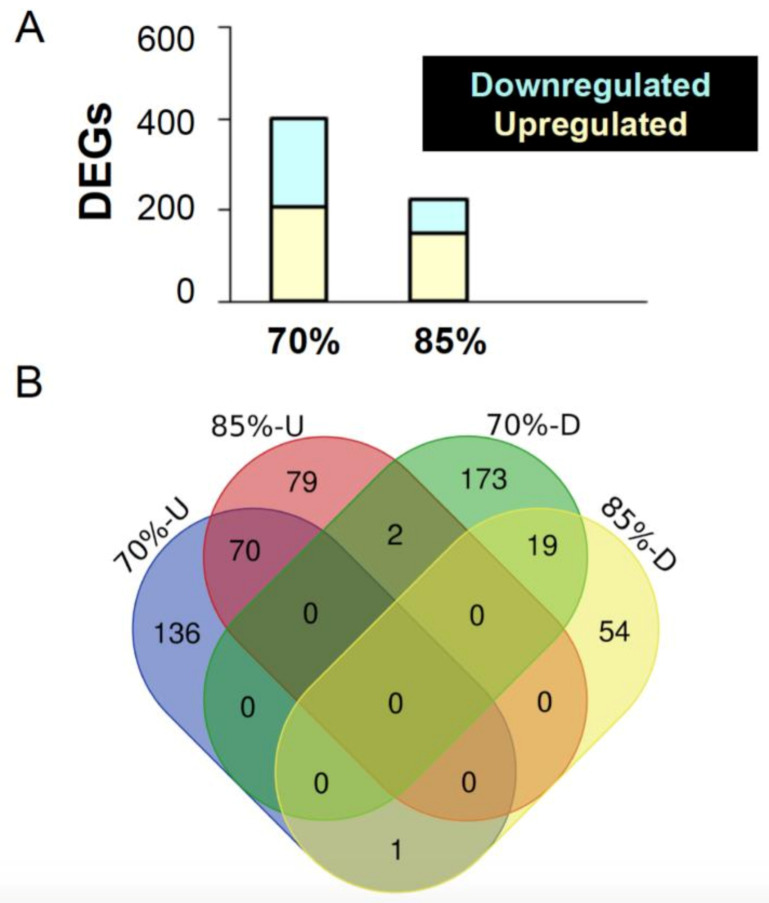
Overview of differentially expressed genes in the mid-intestine of the feed restricted groups (85% and 70%) in comparison to the 100% fed fish. (**A**) The graph shows the number of differentially expressed genes (DEGs) upon 70% and 85% feed restriction in comparison to the 100% fed group. (**B**) Venn diagrams showing the unique differentially expressed genes as well as genes shared between the two feed restricted groups (85% and 70%).

First, we compared the gut gene expression between FR70 and C100 groups (FDR < 0.1). A total of 402 genes showed observable differences in expression (Figure 4). Amongst the 402 genes, 207 were upregulated and 195 were downregulated. When focusing on genes with at least a two-fold difference in expression, we found 343 upregulated and 407 downregulated genes in FR70 zebrafish. 

84.1% of all observed upregulated genes were significantly higher than the control. For example, *Vtg6*, which encode *Vitellogenin 6* proteins that is involved in lipid transporting activity, oestrogen stimulus, and response to oestradiol [38,39] was expressed almost 4.5 times more in FR70 fish. 84.0% of downregulated genes (163/194 genes) were significantly lower in FR70 than C100. For instance, the *tektin 1* (*tekt 1*) gene that encodes for proteins that are involved in cilium assembly [40,41] was downregulated by approximately 3.7 times in FR70 fish.

In contrast with FR70, FR85 zebrafish only had 225 differentially expressed genes relative to controls (FDR < 0.1). Among these 225 genes, 151 (67.1%) were upregulated and 74 (32.9%) were down regulated. 70.2% of downregulated genes and 86.8% of the upregulated genes were significantly modulated by FR85. *tektin 1* was one of the genes commonly significantly downregulated—in fact, it was downregulated even more in FR85 (4.3 times) vs FR70 (3.7 times) fish, as compared to the control. Similar to FR70, *Vitellogenin* 6 (as well as homologs 4, 5, and 7) was significantly upregulated, but only by around three-fold.

We next looked for other genes that were commonly modulated by both FR treatments. Zebrafish in FR70 and FR85 groups shared 70 upregulated genes and 19 downregulated genes (Figure 4). Commonly downregulated genes included *cyp7a1*, which is predicted to have steroid hydrolase activity and to be involved in bile acid biosynthetic process and cholesterol homeostasis, as well as many other predicted functions affecting the endoplasmic reticulum membrane [42,43,44]. *zgc:55461* was another gene that was downregulated in both FR70 and FR85 fish, which is predicted to be involved in microtubule cytoskeleton organization and mitotic cell cycle [45].

Commonly upregulated genes from FR70 and FR85 groups include the *apoa1b* gene, which is predicted to be involved in cholesterol binding, phosphatidylcholine-sterol O-acyltransferase activator, and phospholipid binding activity [46]. Another gene that was upregulated was *c6ast1*. This gene is predicted to be involved in proteolysis and have a metalloendopeptidase activity. 

There were also genes that were differentially regulated between FR treatments, for example, *myhc4,* which is predicted to have ATP binding activity, actin filament binding activity, and motor activity [47]. This gene was upregulated 2.25-fold in FR70, but downregulated 2.49-fold in FR85 (Figure 4). Another example includes *mid1ip1l* gene, which is understood to be involved in the regulation of cytoskeleton organization and regulation of ribonucleoprotein complex localization [48,49,50]. This gene was upregulated 1.08-fold in FR85, but downregulated 1.26-fold in FR70 zebrafish.

### 3.4. Bioinformatics Analysis of RNA-Sequencing Data: GO Pathways-Results (GO)

**Figure 5:**
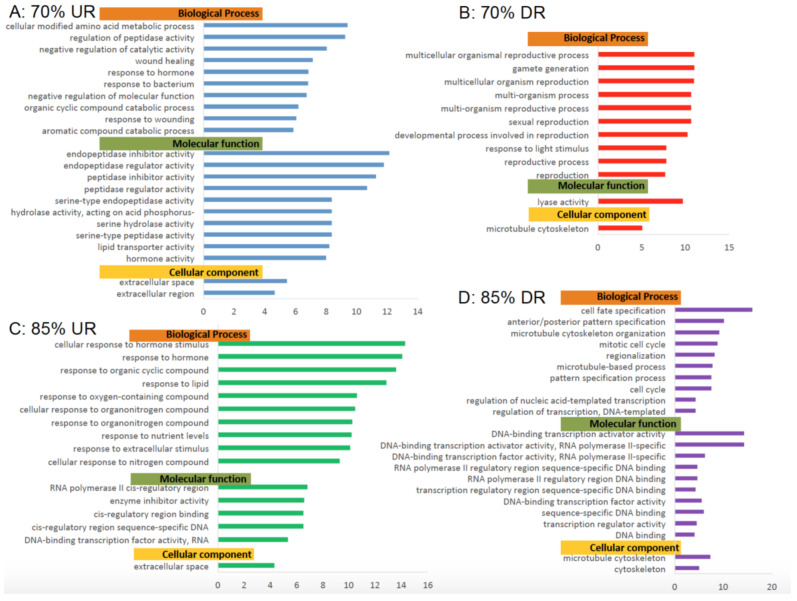
Gene Ontology Analyses of Biological Processes, Molecular Functions and Cellular components. Significantly (false discovery rate (FDR) < 0.05) enriched Gene Ontology (GO) terms from complete analysis for 70% and 85% feeding groups differentially shared genes grouped by biological process, molecular function, and cellular component. GO terms containing a minimum 100 reference genes and a fold change ≥4 or ≤4 are represented. (**A**) 70% upregulated genes; (**B**) 70% downregulated genes; (**C**) 85% upregulated genes; and (**D**) 85% downregulated genes.

Next, Gene Ontology analysis was performed in order to determine the effects of feed restriction on various important pathways in the zebrafish. Zebrafish from FR70 and FR85 were compared to zebrafish from C100. Significantly modulated pathways were classified into three components, (A) Biological Processes, (B) Molecular Functions, and (C) Cellular components (Figure 5).

When comparing gene expression in fish from the FR70 to C100 groups, cellular modified amino acid metabolic processes were upregulated to the greatest extent. Other upregulated biological processes include the regulation of peptidase activity, reduction in catalytic activities, wound healing, and response to bacterium.

Notably, many reproductive pathways, such as gamete reproduction, multicellular organismal reproduction, and sexual reproduction (biological process), were downregulated in FR70 zebrafish. This suppression of reproductive pathways was not observed in FR85 fish. However, pathways from biological processes that were downregulated in both experimental groups included cell cycle pathways, mitotic cell cycles, and microtubule cytoskeleton pathways. Surprisingly, these GO terms were depressed to a greater extent in FR85 fish rather than FR70 fish. The upregulated pathways that were observed in FR85 fish included those involving responses to organic cyclic, lipid, and oxygen containing compounds. Their responses to extracellular stimulus, enzyme inhibiting activity, and DNA-binding transcription factor activity were enhanced within each upregulated pathway.

Notably, genes encoding for extracellular space/region of the cellular components in zebrafish from both experimental groups were upregulated, and more so in FR70 fish as compared to FR85 fish (Figure 5). In contrast, microtubule cytoskeleton components tended to be downregulated by FR.

### 3.5. Bioinformatics Analysis of RNA Sequencing Data: KOG and KEGG Pathways

**Figure 6:**
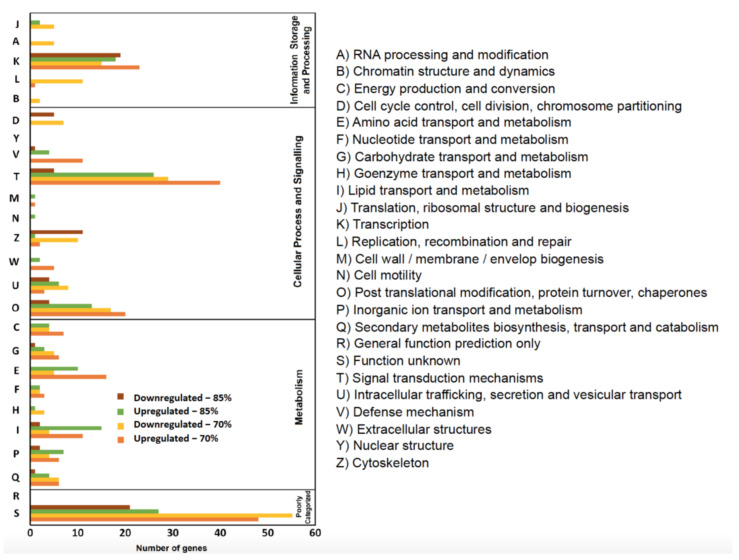
Eukaryotic Orthologous Groups (KOG) functional classification of differentially expressed genes of feed—restricted mid—intestines from two groups were categorized into four main categories: information storage and processing, metabolism, cellular processes, and signaling, and poorly categorized.

The differentially expressed genes from each group were classified into four categories and groups based on their function via KOG annotation. These four categories include: information storage and processing, cellular process and signaling, metabolism, and poorly categorized pathways (Figure 6).

FR led to a predominance of both up- and down-regulated genes within the “cellular process and signaling” category. KOG classification revealed that these genes were mostly related to signal transduction mechanisms (T), with 40 upregulated and 29 downregulated genes for FR70, and 26 upregulated and five downregulated genes for FR85.

In the 70% FR treatment group, 207 up- and 194 down-regulated genes were annotated and classified into metabolism, cellular process and signaling, information storage and processing and poorly characterized categories (upregulated: 26%, 39%, 12%, and 23% and downregulated: 17%, 36%, 19%, and 28%, respectively).

In the 85% FR treatment group, (151 up- and 74 down-regulated genes were annotated and classified into metabolism, cellular process and signaling, information storage and processing, and poorly-characterized categories (upregulated: 31%, 37%, 14%, and 18%, and downregulated: 8%, 39%, 25%, and 28%, respectively).

In addition to signal transduction pathways, other pathways that contain the largest number of differently regulated genes include: transcription pathways (K), post translational modification (O), and amino acid transport pathways (E), and lipid transport and metabolism pathway (I). Carbohydrate transport pathways (G) were more modestly affected (Figure 6).

Consistent with our observation of more upregulated genes, FR generally induced more upregulation when compared to downregulation in most pathways. However, the transcription pathways (K) and cytoskeleton pathway (Z) showed more downregulation in the FR85 treatment. Similarly, for both FR70 and FR85 groups, there were many more downregulated than upregulated genes for cell cycle control, cell division, and chromosome partitioning pathways (D), which is consistent with our GO analysis results (Figure 5).

KEGG pathway analysis was also carried out to evaluate the number of genes in each biological pathway that were up or downregulated (Table 2). Many pathways that were related to metabolism were significantly modulated. Again, in most of these pathways, feed-restricted fish were observed to have more upregulated than down regulated genes relative to the control group. The pathways included basic metabolism (30 genes upregulated, 28 genes downregulated (FR70), 23 genes upregulated, three genes downregulated (FR85)), purine metabolism, carbon metabolism, cholesterol metabolism, and glycolysis/gluconeogenesis.

Examples of upregulated genes that are involved in glucose metabolism include *glucokinase,* which is involved in glucose homeostasis, and type B pancreatic cell development [51,52] and *glucose-6-phosphatase a,* which is predicted to be involved in gluconeogenesis and glucose 6-phosphate metabolic process [53].

## 4. Discussion

This study was designed to investigate the short-term (month long) effects of an 85% or 70% FR protocol on growth, intestinal morphology, and intestinal gene expression in 3 mpf zebrafish (AB strain). Our results generally did not reveal a significant growth difference between the feed-restricted fish and controls, particularly for male fish. However, we did observe increased size heterogeneity in feed-restricted male fish (particularly in the FR70 group), and significant effects of FR on the weights of female fish at certain time points. It is possible that the variable male-female sampling ratios were the cause of the fluctuations that were observed in females’ weights by week and treatment. Intra-species food competition might also explain the size heterogeneity [54,55] during FR.

Our results are consistent with other FR studies in fish. A 2008 study on stickleback (*Gasterosteus aculeatus)* that were fed 2% (vs. 10%) of its body weight concluded that FR did not significantly affect the mean standard length or weight [56]. A 30-day 50% FR experiment that was conducted on European bass (*Dicentrarchus labrax*) concluded that both fish weight and filet yield were not significantly affected by the new feeding protocol [27]. In contrast, calorie or feed restriction studies carried out on mammals, such as mice and monkeys, have found that a restricted diet does indeed significantly reduce their body weights [15,21,22,57].

Fish feed is one of the major expenses to be factored in fish husbandry and it typically represents 50–70% of aquaculture production costs [58]. Although a distant relative from food fish, our zebrafish FR study suggests that mild FR (e.g., 90%) could potentially be a sustainable practice, given the generally subtle effects of 85% FR on overall growth, although factors such as sex also need to be considered.

Our study also reveals a negative impact of FR on gut circumference and a trend towards shorter mucosal height, while other markers, such as muscularis thickness and goblet cell numbers, remained unchanged. A previous study in *Lates calcarifer* had reported a significant reduction of mucosal height, goblet cell numbers, and muscularis thickness upon food deprivation, although circumference was not measured [26]. However, these fish were completely starved. Hence, our results are complementary in showing that even moderate FR can affect some aspects of gut histology. Interestingly, we also observed sex differences in intestinal mucosal height, with male fish having significantly shorter mucosa, which might relate to the other sex differences that were observed in the gut [59].

It is known that FR can affect intestinal mucosal structure and transport function in mammals [60,61]. For example, one study indicated that the crypt depth was significantly lower at both 21 and 27-month-old rats, and the villus height was significantly different in 21-month-old diet restricted rats as compared to the control [62]. Our observed effects of FR on serosa circumference, and possibly also mucosal height, suggests that FR similarly impacts intestinal transport and function in zebrafish. 

The consequences of a reduced intestinal circumference are still not widely reported. However, based on the known functions of the serosa, intestinal mucosa, and muscularis, there is a possibility that metabolic pathways, food absorption, or even the host’s susceptibility to pathogens could be compromised [63,64]. In studies on fruit flies and humans, intestines have been reported to shrink during phases of starvation. However, this process is reversible and repeatable [65]. Hence, the impact of FR on gut circumference could also be a temporary response or physiological adaptation to a reduction in food quantity. 

Beyond gut histology, FR has been reported to influence the expression of genes that are involved in metabolism. For example, a recent study in rats found that glycolytic enzymes, pentose and pyruvate metabolism, oxalate, and urea degradation were significantly more abundant after FR [66]. Our findings are in agreement with these studies, since we observed an upregulation of pyruvate metabolism pathways, insulin secretion, glycolysis, gluconeogenesis, and other genes in carbohydrate transport and metabolism pathways, such as *Ldhbb*, *gck*, and *g6pca.1*, in both 70% and 85% feed restricted fish. Our data complements a study conducted by Casirola, Lan, and Ferraris [15], which observed increased *D*-glucose transport and uptake in feed-restricted mice at all intestinal positions. Similarly, another report found that feed-restricted fish were able to assimilate greater volumes of glucose, fructose, and even proline and glutamine in the proximal small intestine [67].

Casirola, Lan, and Ferraris [15] also observed an increased uptake of amino acids, such as *L*-proline, in the intestines of feed-restricted mice. The transport of both *L*-glutamine and *L*-aspartate were also higher than control mice. Notably, we observed many upregulated genes related to amino acid metabolism, including a 3.52-fold increase in *L*-amino acid peptidase activity in FR70 fish.

Many studies have also identified the downregulation of certain metabolic pathways under calorie or feed restriction conditions. One study reported the downregulation of genes that are involved in glucose and protein metabolism in feed-restricted (60% ration) rats [68]. In fact, one of the genes that was downregulated by more than two-fold was the aldose reductase gene, which is involved in glucose metabolism. Notably, this gene was also downregulated by more than two-fold in the zebrafish gut. Furthermore, in both the above study and ours, cell cycle and cell growth pathways were also downregulated. 

The mechanisms governing FR-induced changes in gut gene expression are unknown. Some evidence suggests that FR, as well as other dietary manipulations, can modulate the gut microbiome [69], which may, in turn, affect gene expression in the host [70]. In fact, our results show that genes involving response to bacteria were upregulated by 70% FR (Figure 5). An example of gut microbiota modulation was shown in a study by Navarro-Barron and colleagues that indicated a greater number of certain bacterial genera detected in the intestines of fat-restricted male and female zebrafish [71]. Another study conducted in 2018 found that Lactobacillus was found to be significantly enriched in feed-restricted rats at all points of the study [66].

Calorie or feed restriction has been proposed to bring about many health benefits, such as a reduced risk for cardiovascular disease, diabetes, and even cancer, as well as boosted immunity [9,12,13,72,73]. A recent study reported that feed restriction increased B cells and T cells in the bone marrow, although they also observed a significant decrease in lymphocyte subsets across the spleen, blood, and pooled brachial [74]. Feed-restricted mice (70% ration) were also observed to have their aging-distributed immune ecosystem favourably reversed [21].

In our FR study, various immune pathways were upregulated, such as wound healing/response to wounding, and response to bacterium, although it is unclear whether this is an indication of cellular/oxidative damage or a beneficial response. Previous studies indicate that a short-term FR reduced the mortality of mice injected with *Salmonella* bacteria [75]. On the other hand, long term FR experiments concluded that, although young mice under FR are protected against oxidative stress, their results also show a delayed maturation of macrophage function, which increases their susceptibility to bacterial infection. Chronic FR was also observed to have a positive impact on wound healing especially in older animals [76]. FR also reduces disease susceptibility in catfish, as mentioned in the introduction [4,5,6,7].

On the contrary, it has been observed that short term overfeeding of zebrafish with a high fat diet led to metabolic alterations, such as hyperglycemia and ectopic lipid accumulation in the liver [77]. Another study on overfeeding in rainbow trout observed the development of immunological disorders and increased susceptibility to infectious diseases [78]. In aquaculture, fish with better immunity and health would be preferred, as they would be more resistant to disease outbreaks, protecting the farmers from substantial loss. Our study may also inform medical research as to the immune-related benefits of restricted feed or caloric intake.

Although there might be advantages for using a feed-restricted diet, there are also possible downsides. In our study, we found that gamete reproduction and sexual reproduction pathways were downregulated in FR70 zebrafish by at least 10 times for each of the 140 genes in each pathway, although it was not observed in FR85 zebrafish. A similar report carried out by Mattison (2013) indicated that the reproductive maturation of prepubescent monkeys was also delayed when they were exposed to 70% feed restriction in their early life. Hormonal changes in aging female monkeys were significantly decreased, along with number and lengths of menstrual cycle [20]. This is a critical disadvantage, as hormonal changes could be accompanied by skin and body composition changes, cognitive impairment, or even the loss of bone mass in humans [20]. Interestingly, in zebrafish, the study that was carried out by Leibold and Hammerschmidt [30]) revealed that reproduction was, in fact, prioritized over somatic growth, albeit only in adult (9–12 month olds) that had reached maximal body mass. Hence, the developmental stage of a vertebrate should be taken into consideration when implementing metabolic interventions or FR studies.

The detection of reproductive transcripts in our analysis is potentially puzzling. It is possible that the transcripts that were measured in our study may not have been solely derived from the zebrafish guts. Because of the small size of the animal, the position of the zebrafish’s gonad and intestines are very close to one another, raising the possibility of some contamination. Alternatively, there are genes with multiple functions that are involved in digestive and reproductive pathways, such as *zgc:56699*, *acsl4b,* and *Cldng,* which could have contributed to our observations of reproductive gene downregulation. 

Our study also revealed the upregulation of genes encoding extracellular space proteins. Previous studies have discussed the importance and benefits of an extracellular matrix (ECM). The ECM interacts with cells to regulate diverse functions, including proliferation, migration, and differentiation. ECM remodeling is also crucial for regulating the morphogenesis of the intestine and lungs, as well as of the mammary and submandibular glands [79]. ECM has also been thought to be an ideal therapeutic substrate for functional remodeling of damaged gastrointestinal (GI) tissue, owing to its composition and roles in tissue development, wound repair, homeostasis, and also to help modulate the innate immune system [80]. These conclusions are closely related to our observations regarding the upregulation of wound healing and immune system pathways.

In conclusion, we have shown that moderate FR does not drastically affect the zebrafish’s growth and it could even potentially bring about certain health benefits. However, further investigation regarding its functional impact on the histology and physiology of the gastrointestinal tract, as well as other organs, will be needed in order to further assess the effects of FR on zebrafish. Note that, although the weight profile and histological data point to certain sex dependent differences between the males and females, the RNA-Seq analysis was performed using a mixture of males and females. Hence, a limitation of the study is the inability to glean sex-specific transcriptomic differences that are induced by feed restriction. In addition, further studies are needed to address the detriments caused by FR, such as delayed sexual maturation, which might be undesirable under certain conditions. Overall, our study complements the expanding literature regarding the potential effects of feed or caloric restriction on metabolism and health across vertebrate species.

## Figures and Tables

**Table 1:** The number of male and females represented in the three treatment groups at weekly intervals.

	Group 3 (100% Feeding)		Group 2 (85% Feeding)		Group 1 (70% Feeding)	
Week	Male	Female	Male	Female	Male	Female
0	19	11	NA	NA	NA	NA
1	25	5	20	10	20	10
2	23	7	25	8	18	12
3	18	12	14	16	20	10
4	20	10	20	8	18	7

**Table 2:** Significant (*p* < 0.05) pathways characterized by Kyoto Encyclopedia of Genes and Genomes (KEGG) analysis of differentially expressed genes from 70% and 85% FR groups. The numbers of genes mapped to annotated pathways are provided.

Pathway	Number of High/Low Abundance Genes in FR70	Number of High/Low Abundance Genes in FR85
map01100 Metabolic pathways	30/28	23/3
map04024 cAMP signalling pathway	6/1	5/1
map00230 Purine metabolism	5/1	2/0
map01200 Carbon metabolism	5/3	2/0
map04979 Cholesterol metabolism	5/5	2/1
map04911 Insulin secretion	4/1	3/0
map04978 Mineral absorption	4/2	3/0
map00010 Glycolysis / Gluconeogenesis	4/2	1/0
map00630 Glyoxylate and dicarboxylate metabolism	4/2	2/0
map04218 Cellular senescence	3/5	4/1
map04923 Regulation of lipolysis in adipocytes	3/0	2/0
map00620 Pyruvate metabolism	3/2	0/0
map04810 Regulation of actin cytoskeleton	3/0	4/1
map04510 Focal adhesion	2/0	2/1
map04210 Apoptosis	2/1	3/2
map04973 Carbohydrate digestion and absorption	2/1	2/0
map04975 Fat digestion and absorption	2/3	4/0
map05210 Colorectal cancer	2/3	4/2
map04977 Vitamin digestion and absorption	2/1	3/0
map04217 Necroptosis	2/1	0/3
map01230 Biosynthesis of amino acids	1/3	3/0

## Data Availability

The sequences have been submitted to NCBI under BioProject accession number PRJNA695140.

## Supplementary Materials

The following are available online at https://www.mdpi.com/1422-0067/22/4/1814/s1, Figure S1: Histological analyses of feed-restricted zebrafish by sex., Table S1: Summary statistics of the RNA-Seq data and mapping rates to the zebrafish reference genome.
