# Supplementary material for: Feed Restriction Modulates Growth, Gut Morphology and Gene Expression in Zebrafish
Source: Int J Mol Sci. 2021 Feb 11;22(4):1814. doi: 10.3390/ijms22041814 (PMC7917766; doi:10.3390/ijms22041814)
Supplement: Supplementary file 1 [file ijms-22-01814-s001.zip › Supplementary files to upload Final version/Supplementary Figure S1.docx]

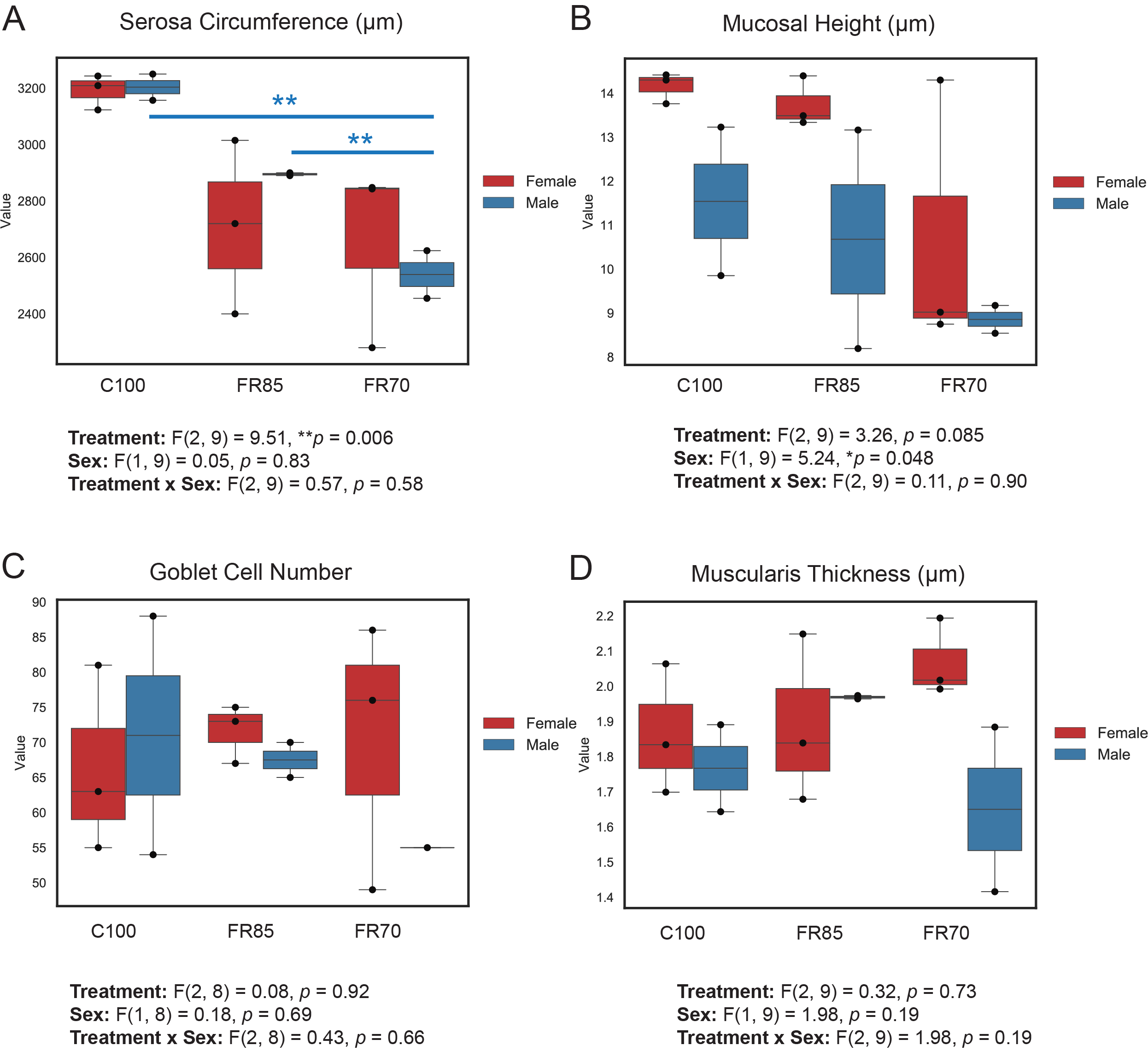


**Figure S1. Histological analyses of feed-restricted zebrafish by sex. (A)** External circumference of serosa, **(B)** mucosal height **(C)** goblet cell number **(D)** muscularis layer thickness were measured from sections of the midgut, and divided according to sex. Each dot refers to the average data from a single fish. The box plot shows median, interquartile interval, and data range excluding outliers; each dot represents average values from an individual fish. Two-way ANOVA was performed between the mean of each group with the mean of the control group, followed by the Tukey Post-hoc test, adjusting for multiple comparisons. Means significantly different from Group 100% are noted with —*Padj<0.05, **Padj<0.01
